# Reaching hidden youth in Singapore through the Hidden Youth Intervention Program: A biopsychosocial approach integrating mental health and social work interventions

**DOI:** 10.3389/fpsyt.2023.1133659

**Published:** 2023-03-16

**Authors:** Sonia Khiatani, Denise Liu, Benjamin Sen Son Yeo, John Chee Meng Wong

**Affiliations:** ^1^Department of Psychological Medicine, National University Hospital, Singapore, Singapore; ^2^Strategic Planning and Research Department, Fei Yue Community Services, Singapore, Singapore; ^3^Youth Services, Fei Yue Community Services, Singapore, Singapore; ^4^Department of Psychological Medicine, Yong Loo Lin School of Medicine, National University of Singapore, Singapore, Singapore

**Keywords:** hikikomori (social withdrawal), hidden youth, Singapore region, multidisciplinary, intervention, biopsychosocial, socially withdrawn behavior

## Abstract

Hidden youth are youth who withdraw from society for at least 6 months, physically isolating within their homes or rooms. There has been a steady rise in this phenomenon across many developed countries, and this trend is expected to continue. As hidden youths often present with complex psychopathology and psychosocial issues, multi-factorial intervention approaches are recommended. To reach this isolated population and address gaps in services, a community mental health service and a youth social work team collaborated to develop the first specialized intervention for hidden youth in Singapore. This pilot intervention combines components from Hikikomori treatment models from Japan and Hong Kong, and a treatment program for isolated individuals diagnosed with Internet Gaming Disorder. This paper describes the development of the pilot intervention model- a four-stage biopsychosocial intervention targeting the complex needs of hidden youth and their families- and illustrates its implementation and challenges faced through a case study. Based on 2 years of service delivery to 25 youths, good practices such as utilizing novel outreach strategies and the importance of involving and caring for caregivers are also highlighted. Preliminary outcomes of this ongoing pilot intervention indicate reductions in social withdrawal behavior and increased engagement in school or work, especially for youth at the final stage of intervention. Strengths of the program include its multi-disciplinary and flexible nature, and the whole-family approach. Limitations of this program included a lack of information on Singaporean hidden youth and the lack of quantitative outcome data of this pilot program. In future, we aim to further enhance program elements through collaboration with international and local partners, and to develop an evaluative framework to determine program effectiveness.

## 1. Introduction

Hikikomori, or hidden youth, are youth who have isolated themselves in their room or their homes for a period of 6 months (1, 2), withdrawing from face-to-face social contact and participation in school or employment (3). There is currently no prevalence data available in Singapore, however, estimates from Japan (4) and Hong Kong (5) indicate that the prevalence of hikikomori is between 1.2 and 1.9%. As recent studies have shown that hikikomori risk is associated with more time spent in lockdown during the COVID-19 pandemic and increased internet use (6), it is possible that the current prevalence is even higher.

Youth who experience extensive social isolation are more likely to have poorer physical health (7), conflictual family relationships (8), increased risk of suicidal behaviors (9), and poorer psychological outcomes (10). Though mental illness is not the primary cause of social withdrawal (11), hidden youth are at a higher risk of presenting with co-morbid psychiatric disorders such as social anxiety and depression (12).

Hidden youths also face stigma as they fail to conform to social norms of the competitive Singapore society which focuses on academic and career achievement (13). In turn, caregivers experience a high burden of care and pressure from others to reintegrate their youths into society (14). Hidden youth who face rejection in mainstream society often form identities and support systems within online communities, and according to a study in Hong Kong, their quality of life improves as the duration of their withdrawal increases (15). It is thus not surprising that most hidden youth do not seek help independently (16).

The understanding of underlying psychopathology of hikikomori is heterogeneous and differs within the region (17). As there are no studies of hidden youth in Singapore, our understanding is based on models from other countries and case studies seen by professionals in Singapore. In Japan, hikikomori is viewed more through the lens of a psychiatric disorder (18), whereas in Hong Kong and Korea, it is seen as a multifaceted social phenomenon, which may involve psychological factors (19), or gaming addiction (20). In Singapore, hidden youth behavior is understood to be a complex, multi-factorial phenomenon with individuals presenting with multiple family and socio-cultural issues, and co-morbidities such as gaming addiction or other mental health issues (2). Due to the strong focus on achieving high levels of education for all, and compulsory military service for males at age 18, it is may also be easier to identify socially withdrawn youth in Singapore, creating opportunities for early intervention.

## 2. Program design

Overall, there is a dearth of evidence-based practices for the treatment of hidden youth and there are no programs in Singapore to our knowledge that focus on hidden youths specifically. Services targeting socially isolated youth in Singapore include school-based reintegration programs, mental health counseling, and community-based parenting and family support. Hidden youth are typically not enrolled in these services as programs require at least one engagement with the youth before they can be accepted into the service, which youths typically resist. Service eligibility criteria, such as needing to be enrolled in school to access school-based interventions, also limit access to services. Lastly, most services are time-limited and lack the continuum of care necessary to provide effective intervention to hidden youth. For example, school-based programs usually have a maximum duration of 12 months, and cases are closed after repeated unsuccessful attempts to engage the youth.

To address these gaps in services, we developed the Hidden Youth Intervention Program (HYIP) based on intervention components and effective practices identified from a review of the emerging evidence and treatment models for hidden youth. HYIP combines the expertise of a community-based mental health service, the Response, Early Intervention and Assessment of Community Mental Health (REACH) team, and the Hidden Youth Outreach Service (HYOS) by a social service agency, Fei Yue Community Services.

HYIP adopts a four-stage intervention approach. Our target clients are hidden youth 12–25 years old and their caregivers. Based on information gathered from interviews with caregivers, youth are assessed as being hidden youth if they had isolated at home (not leaving the home more than three times a week) for 6 months or more, resulting in significant functional impairment (3). Youths view this as ego-syntonic. Youths who were isolated between 3 and 6 months were considered pre-hikikomori. Hidden youth were categorized according to severity in three categories- mild, moderate, severe- according to an adapted classification system from Japan (2, 21). Social workers classified the youth based on information from interviews with their caregivers. Youths in the “mild” range are still able to leave their house at times (up to three times a week) though they refrain from social interactions with others; youths in the “moderate” range generally do not leave their houses but maintain some contact with family members; and youths in the severe range, stay within their rooms mostly and do not interact with family members.

### 2.1. A multidisciplinary, multilevel approach customized to severity of isolation

As most effective hidden youth interventions adopt a multidisciplinary approach (22) involving professionals such as psychologists, social workers, and physicians (11), HYIP also involves a multidisciplinary team of psychiatrists and psychologists from REACH who provide mental health assessments and interventions, and HYOS social workers who provide outreach and support to hidden youth and their caregivers.

According to previous studies, the type of intervention should take into account the youth's level of social isolation (11). For example, in an approach utilized in Hong Kong, the extent of social withdrawal determines the entry point for intervention- the most withdrawn youths receive therapeutic intervention, while less withdrawn youth receive educational or social interventions (23). Especially in the early stages of HYIP, intervention is tailored to the severity and extent of social withdrawal behavior. The most withdrawn youth may require intensive outreach and engagement from social workers before they are willing and ready to receive psychological intervention. In contrast, less socially isolated youth who do not present with comorbidities may not require psychological intervention.

### 2.2. Intervention modalities

Home visits conducted by trained professionals (24) are an effective method to engage the youth and family. Evidence has also shown that home visitations which focus on providing supportive services to hikikomori and their families (25) enable them to become more socially connected. Animal Assisted Therapy has also effectively been incorporated into intervention models, and has qualitatively been shown to be effective (2). Online psychological interventions or counseling (22), and game-based interventions utilizing play therapy (26) are shown to be effective in reducing social withdrawal, while integrated counseling, which combines online and offline counseling, is also effective in improving quality of life and wellbeing (27). Similarly, HYIP utilizes a range of intervention modalities at different stages of intervention according to the youth's level of social isolation. For example, online engagement or interventions may be utilized for youth with a high degree of social isolation, while youth who are less socially isolated may receive a combination of face-to-face and online sessions.

### 2.3. Theoretical frameworks and intervention components

**Figure 1:**
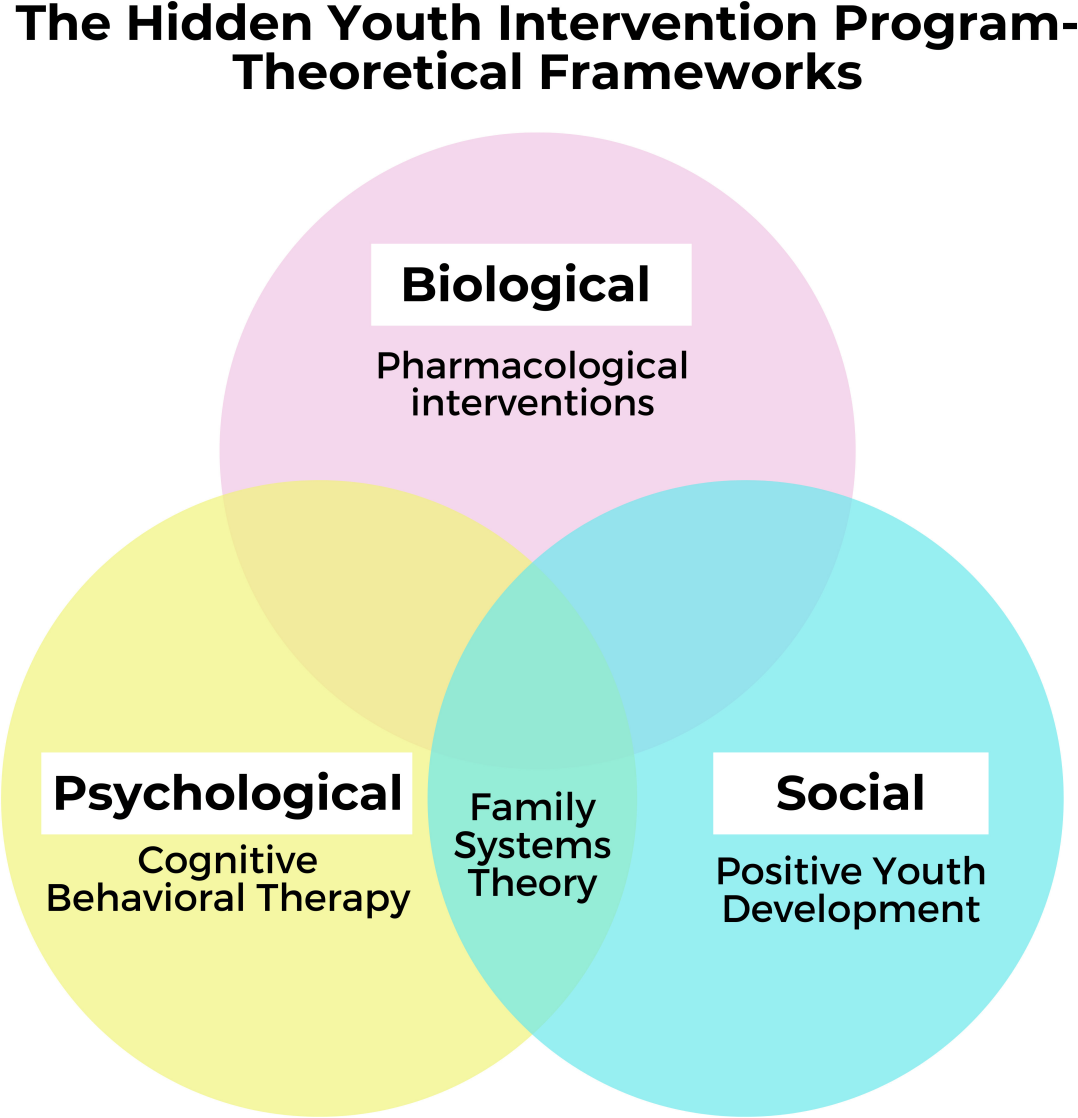
Biopsychosocial model of intervention.

The HYIP intervention model adopts a biopsychosocial approach integrating intervention components from established psychological treatment programs and social work interventions (refer to Figure 1). Biopsycho integrative approaches have been used in hidden youth interventions in Japan (3) and Korea (24), and the psychosocial approach has been used in Hong Kong (28). To address the biological factors contributing to hidden youth behavior, HYIP may involve psychiatric and pharmacological interventions to manage comorbid mental health conditions and address sleep difficulties, which are prevalent among hidden youth (7).

The psychological intervention approach used was that of Cognitive Behavioral Therapy (CBT), due to its established validity in treating comorbidities such as depression and anxiety. CBT-based interventions also target youths' negative beliefs such as the belief that they do not fit into society, or the world is against them (29). HYIP also includes components from PIPATIC,^1^ a CBT-based treatment program (30) targeting internet gaming addiction, which 65% of hidden youth are at risk of or meet the criteria for (24). PIPATIC has been effective in reducing addiction and ensuring reintegration into the community in patients diagnosed with internet gaming disorder (31, 32).

Social work interventions are informed by Family Systems Theory (33), and the Positive Youth Development (PYD) framework (34). According to FST, patterns of interaction in the family system can perpetuate problem behaviors (33). For example, anxious caregivers who express helplessness with their child's behavior and avoid confronting or disciplining their child can perpetuate their child's social withdrawal (35). In line with FST, family interventions to improve parent-child relationships (11), family psychotherapy (18), and parent support groups (22), have been identified as essential components of hidden youth interventions.

PYD is an ecological, strengths-based approach to youth work (34). Research has shown that interventions which develop the 5Cs (Confidence, Connection, Competence, Compassion, and Character) have a positive effect on youth's academic achievement and psychological adjustment (36, 37). According to previous studies, interest-based activities customized to the youth's level of engagement (22, 29) promote positive youth development and social skills (38) in hidden youth. HYIP incorporates interest-based activities to build the youth's Confidence, and Connections with social workers, family members and peers. Job-training (22), vocational skills and reengagement with education (39), which are components of effective hidden youth interventions, also build the youth's academic and vocational competence.

### 2.4. Program implementation

The HYIP pilot began in 2021 with a team of 14 social workers and 1 program manager. Social workers manage 1 to 5 HYIP cases in addition to their other cases. HYIP social workers attended 6 days of training to equip them with knowledge about hidden youth behavior, intervention strategies, engagement skills, and an understanding of youths' psychosocial needs. Five supervisors also provide monthly supervision to monitor the progress of cases and provide support for challenging cases. An external consultant who has extensive experience working with hidden youth was also engaged to develop social workers' skills in providing family intervention.

## 3. Methodology

To describe the HYIP participants and detail their progress, demographic information, case characteristics, and social workers' assessments of the youth were compiled from administrative data. We conducted descriptive analyses, reporting data only in aggregates such as means and standard deviations.

In October 2021, three reflective practice circles involving 14 social workers were conducted to understand the challenges social workers faced, and identify good practices utilized during HYIP. Reflective practice groups or circles involve social workers reflecting on their practices in a group setting to understand factors contributing to case outcomes (40), for the purpose of mutual learning and support, and practice improvement (41, 42). As the reflective practice circles were conducted as part of program improvement, deidentified, anonymized transcripts of the sessions were made available to the authors as secondary data for the purpose of collating learning points and good practices. Deidentified transcripts were coded using the six-step process of thematic analysis (43), and data were stored and coded using NVivo 11. Primary cycle coding involved reading each transcript in detail and developing initial codes inductively. Initial codes were combined to form overarching themes in subsequent rounds of coding. Identified themes relevant to the description of HYIP, such as challenges and good practices, are described at each stage of the intervention and the Discussion section.

The Fei Yue Ethics Advisory Committee reviewed this study (refer to Ethics Statement) and determined that administrative data and deidentified transcripts constituted secondary data collected for the purposes of program improvement.

## 4. The Hidden Youth Intervention Program

**Figure 2:**
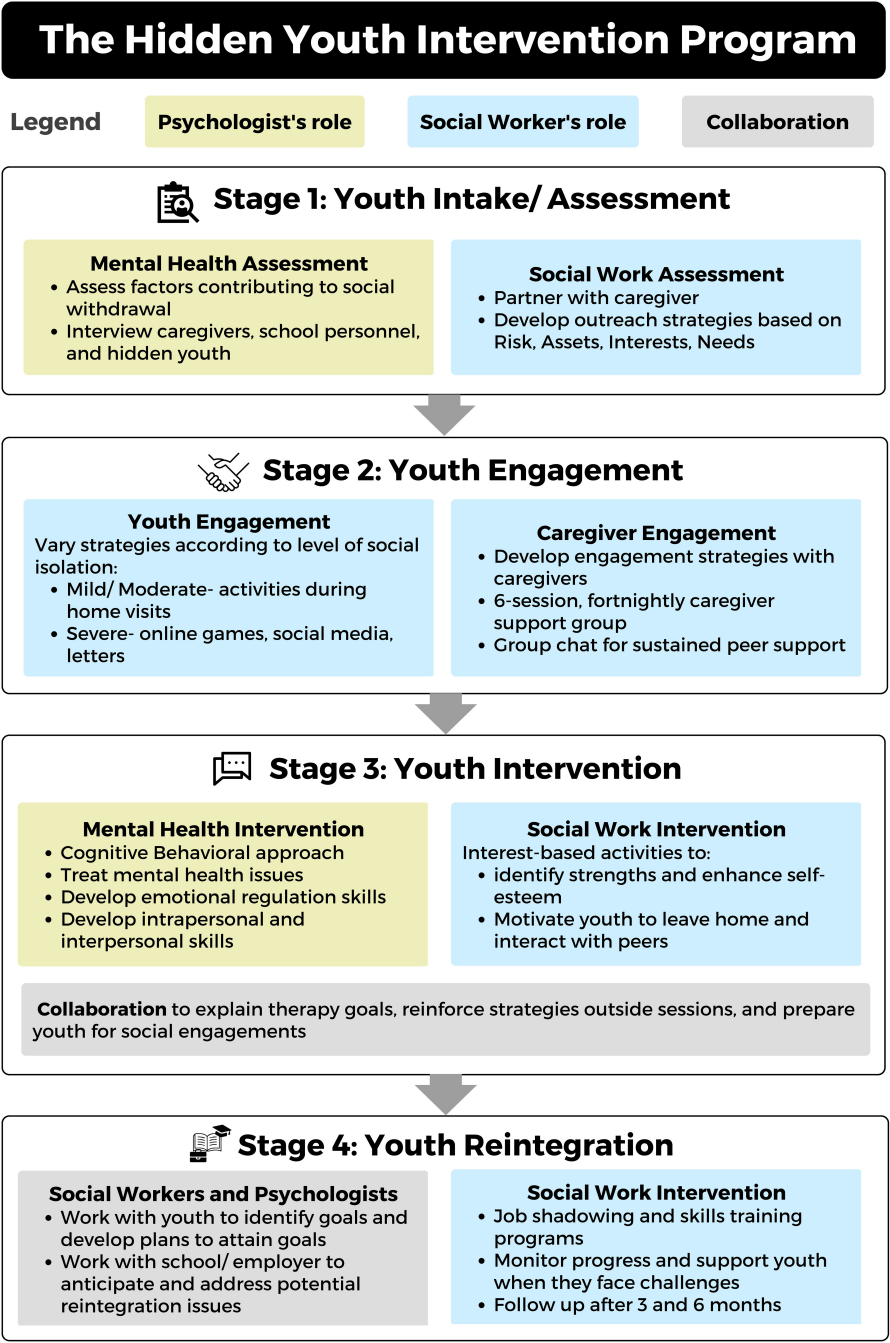
The Hidden Youth Intervention Program: Stages of intervention and best practices.

The four stages of HYIP are summarized in Figure 2.

### 4.1. Stage 1: Youth intake/assessment

Upon referral from schools, parents or community partners, youths are assessed online or through home visits instead of at the school or clinic. Home visits allow workers to engage the youth and assess their living environment, while video or phone interviews may be the only way to engage hidden youth who decline face-to-face contact.

#### 4.1.1. Mental health assessment

This focuses on the biological and psychological factors that contribute to hidden youth behavior. Detailed information about the youth's developmental history and family background is gathered from interviews with caregivers and school personnel. If possible, the youth is also interviewed, or given self-report measures to complete such as the Hikikomori Questionnaire 25 (44), the Beck's Youth Inventory (45) to screen for depressive, anxiety, and anger symptoms, and the Internet Gaming Disorder Scale (46). However, due to the reclusive nature of the youths, it was only possible for hidden youths of “mild” severity to complete the self-report measures. The mental health case formulation considers predisposing factors such as the youth's neurodevelopmental history, precipitating factors such as academic failure or interpersonal conflict, and perpetuating factors such as gaming addiction and poor coping styles. Other biological factors assessed include the youth's sleep, appetite, and medical history.

#### 4.1.2. Social assessment

Social workers partner with the caregiver to understand the youth's social withdrawal behaviors and develop outreach strategies using the R.A.I.N (Risk, Assets, Interests, and Needs) Framework (47). The R.A.I.N. framework combines established youth intervention models such as the Risk-Need-Responsivity model (48, 49) and the Positive Youth Development approach (34).

#### 4.1.3. Challenges and good practices

As informants are often not fully aware of the youth's issues due to their withdrawn nature, it is difficult to accurately conceptualize the case and develop an effective intervention plan. Therefore, case formulation at this stage is preliminary, and assessment is an ongoing process. Regular case conferences between the mental health and outreach service are conducted to provide updates about the latest case conceptualization.

Providing psychoeducation to caregivers about hidden youth is essential in Stage 1. Social workers face challenges regulating caregivers' anxiety, anger, and grief regarding their child's withdrawal. Empathizing with caregivers, attending to their anxiety, and working with them to process their emotions enabled social workers to engage caregivers effectively. Social workers also face challenges managing caregivers' expectations about the expected timeline for progress to occur. Informing the caregivers upfront about the approach to intervention, including the expected duration required to engage youths (up to 12 months), the overall timeline of the intervention, the frequency of home visits, and the need to respect the boundaries set by the youth reduced the likelihood that caregivers would pressure the youth to meet the social worker in subsequent stages of intervention, and empowered caregivers to manage the expectations of external parties such as other family members and the school.

### 4.2. Stage 2: Youth engagement

This stage—the most challenging component of the intervention- involves engaging the hidden youth and family. Social workers spearhead this stage while consulting mental health professionals where necessary.

#### 4.2.1. Engaging youth

Engagement strategies vary according to the youth's level of social isolation. Youths who are severely isolated could be engaged through online games or social media platforms, while youths who are less isolated might immediately engage with the social worker during home visits. For example, social workers interact with youths through activities they enjoy, such watching their favorite television shows, baking, fishing, or even visiting animal shelters together.

Social workers need to demonstrate creativity and sincerity for socially isolated youths to be willing to connect with them. Some strategies include writing letters to the youth, delivering their favorite food, and making regular home-visits despite the youth's lack of response. Persistent and patient “one-way” engagement initiated by the social worker enable youth to be in control of the interaction and respond only when they feel comfortable doing so by, for example, responding to letters.

Once initial contact is established, social workers must remain flexible and attuned to the youth's need for safety and control. For instance, some youths are not comfortable with the social worker entering their rooms, which they view as their safe space. Others are concerned that family members would overhear their conversations. Social workers give youths the autonomy to decide when and where they would like to engage: at home, outside, or through online platforms such as Zoom or Discord, and their preferred timing. Workers also avoid discussing topics that might cause discomfort such as school or work, instead engaging youths through casual activities, such as watching TV together or playing games with forfeits such as going outside to smell the neighbor's shoes!

#### 4.2.2. Engaging caregivers

Caregivers often feel helpless, angry, or ashamed when their child remains socially withdrawn despite their best efforts. These emotions often affect their interactions with their child, possibly reinforcing their child's social withdrawal. In Stage 2, caregivers are encouraged to participate in a 6-session, fortnightly caregiver support group facilitated by psychologists and social workers which covers topics such as mental health, parenting styles, attachment, and communication. This group allows caregivers to connect with each other and share their difficulties and successes. Caregivers are also encouraged to sustain contact through a group chat moderated by facilitators. Social workers also aim to empower caregivers and work in partnership with them. Good practices include recognizing caregivers as the experts of their child's life, working with caregivers to co-develop engagement strategies based on caregivers' understanding of their child, and obtaining regular feedback about the child's progress.

### 4.3. Stage 3: Youth intervention

#### 4.3.1. Mental health intervention

Stage 3 targets the development of new skills and treatment for any mental health issues. Youths transition to Stage 3 once there is increased comfort in regular activities and increased engagement with family members and their social worker. Skills that may be covered include:

a) Cognitive Behavior skills (e.g., thought identification, cognitive restructuring, behavioral experiments, and exposure).
b) Emotional Regulation skills (e.g., emotional awareness and literacy; strategies to self-regulate in response to intense emotions).
c) Intrapersonal skills (e.g., mindfulness, identification of strengths and values).
d) Interpersonal skills (e.g., social and communication skills, including non-verbal and verbal communication, and social problem-solving skills).

These skills need not be taught in order but may vary depending on the youth's individual goals, strengths, and weaknesses. Youths presenting with mental health issues are encouraged to seek psychological and/or psychiatric help. The methodological approach to therapy is determined by the psychologist based on the mental health needs of the youth as well as the best evidence base. Sessions may be conducted online initially until the youth is comfortable to engage face-to-face. The youth's psychologist also works closely with the social worker to: (a) explain the goals of therapy to the youth and family; (b) discuss how the social worker can reinforce strategies learned outside of therapy sessions; and (c) jointly prepare the youth for upcoming social engagements. The psychologist also updates the family about the youth's progress and encourages family members to reinforce intervention strategies in the home setting.

#### 4.3.2. Social work intervention

Once trust has been established, the social worker will introduce suitable interest-based activities such as baking and animal-assisted activities. The goals of these activities are to motivate youths to leave their homes, engage with others, and develop social and emotional regulation skills. Social workers can also use these activities to observe the youth's communication, behavior, and strengths, and work with youths to translate these strengths to other aspects of their lives. Through interest-based activities, youths can also learn vocational skills that enhance their sense of competency and self-esteem, which are vital to reintegration (14).

#### 4.3.3. Challenges and good practices

Social workers face challenges remaining attuned to the youth's comfort level and pacing sessions accordingly, and motivating youth to engage with others. Social workers must be cognizant of how engagement is paced and observant of the youth's reactions to ensure that sessions stay within the youth's level of tolerance. Workers also intentionally step back once they sense that youths are not comfortable with the level of contact. For example, when a youth stopped responding to the worker, the worker reverted to “one-way” forms of engagement such as delivering food and writing notes to show his concern instead of texting or calling. When working with a youth to accomplish a goal that he set out to achieve- engaging in an activity outside of his house—another worker discussed the timeline and targets the youth was comfortable with to ensure that the action plan stayed within the youth's comfort zone.

### 4.4. Stage 4: Youth reintegration

In Stage 4, youths gain independence and reintegrate back into school, employment, and their communities. HYIP professionals work with youths to identify their goals for the future and develop concrete plans to attain these goals. For example, youths who are interested in working in specific industries are referred to skills-training and job shadowing programs run by youth workers from the same organization (Fei Yue Community Services). Social workers work closely with program staff to monitor youths' progress and support them when they encounter challenges. For youths who choose to return to school, social workers and psychologists work closely with school personnel to anticipate and address potential school integration challenges.

There are periodic follow-ups by HYIP professionals at the 3- and 6-month point after the end of the program to ensure that treatment gains are sustained.

## 5. Case study

**Figure 3:**
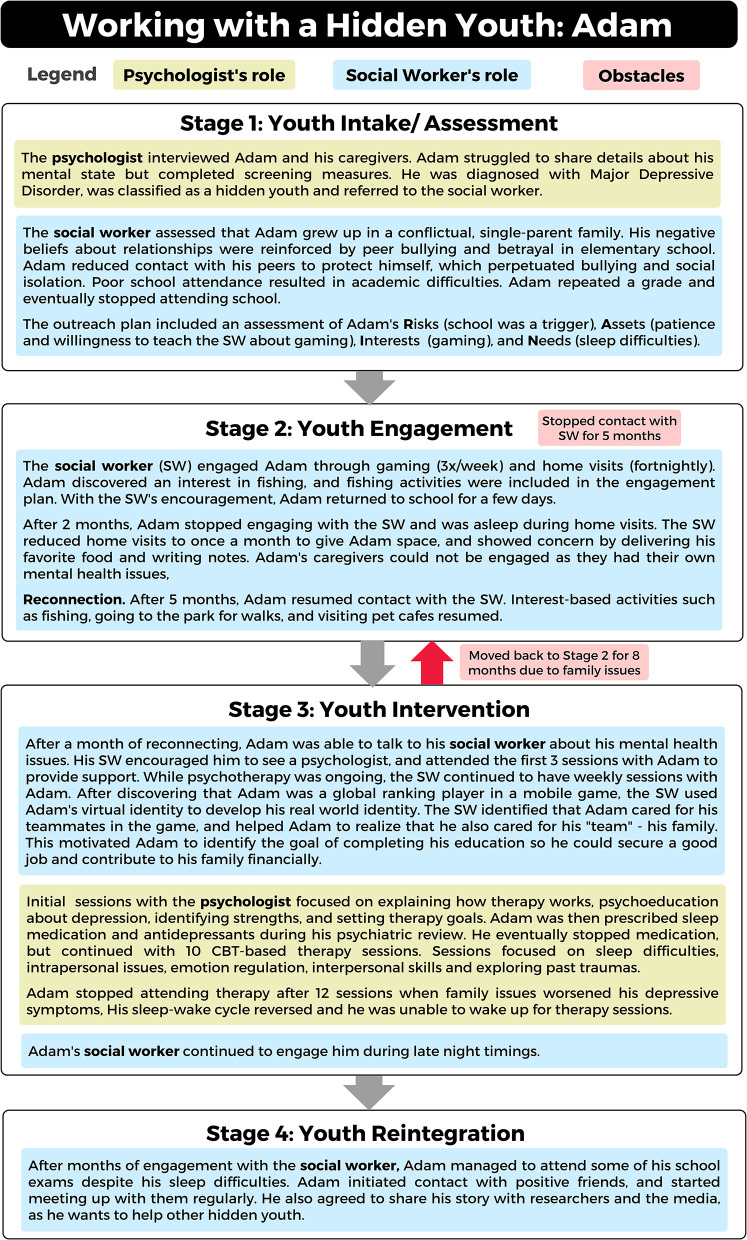
Working with a hidden youth: a case study of Adam.

Adam's experience in HYIP is used to illustrate the 4-step intervention model. Adam is a 15-year-old living with his aunts and older cousin. He was referred to HYIP by his school for concerns about long-term absenteeism. Please refer to Figure 3 for Adam's case study and his journey through HYIP.

## 6. Case characteristics and initial outcomes

Since 2021, HYIP has served 25 youth and their families. As HYIP is still ongoing, only 2 cases have been closed so far. At intake, youth were on average 15.58 (*SD* = 1.65) years old. The average age of onset of social withdrawal was 13.52 (*SD* = 1.45), and the average delay between onset of social withdrawal and intake was 2.06 years (*SD* = 1.32) which is relatively shorter compared to data from Japan [4.4 years, (50)]. Youth served by HYIP are similar to the typical profile of hidden youth −64% were male, and 72% were from middle-class families (16).

As illustrated by Table 1, which details case characteristics such as duration of intervention, HYIP is an intensive intervention requiring substantial resources and manpower. On average, cases at the reintegration stage have been open for 14.11 months, with an average of 26 sessions conducted by social workers. Preliminary outcomes are presented in Table 2, which includes a comparison of severity ratings (mild, moderate, and severe) at intake and the latest point of contact. Overall, 56% of youth showed reductions in level of severity (e.g., from moderate to mild) between intake and the latest point of contact. Intervention stage varied with outcomes, with only 1 out of 5 youth (20%) at Stage 1 demonstrating reductions in severity, compared to 5 out of 6 youth (83%) at Stage 4.

**Table 1:** Case characteristics (*n* = 25).

	**No. of youth**	**Intervention duration in months**		**No. of sessions**	
	**No (%)**	**Mean (SD)**	**Range**	**Mean (SD)**	**Range**
Stage 1	5 (20%)	8.93 (4.91)	2.93–13.23	8.20 (6.57)	3–10
Stage 2^a^	10 (40%)	9.97 (4.77)	4.70–20.97	9.80 (4.85)	4–16
Stage 3	4 (16%)	10.92 (1.91)	8.80–13.30	38.00 (17.57)	18–59
Stage 4	6 (24%)	14.11 (5.75)	6.47–24.90	26.00 (13.80)	9–44
**All**	**25**	**10.80 (5.14)**	**2.93–24.90**	**17.88 (15.00)**	**3–50**

^a^Includes 2 cases closed unsuccessfully.

**Table 2:** Case progress at each stage of intervention (*N* = 25).

	**Severity at intake**			**Current severity**				**Changes in severity**		**Engaged in school/work**	
	**Mild**	**Mod**	**Severe**	**None** ^a^	**Mild**	**Mod**	**Severe**	**No change**	**Reduced**	**No**	**Yes**
Stage 1	0	4	1	0	1	3	1	4	1	4	1
Stage 2^b^	0	5	5	0	6	3	1	3	7	6	4
Stage 3	0	3	1	0	1	2	1	3	1	4	0
Stage 4	2	4	0	1	5	0	0	1	5	1	5
**All**	**2**	**16**	**7**	**1**	**13**	**8**	**3**	**11**	**14**	**15**	**10**
**%**	**8%**	**64%**	**28%**	**4%**	**52%**	**32%**	**12%**	**44%**	**56%**	**60%**	**40%**

^a^Youth does not exhibit hidden youth behavior/social withdrawal.

^b^Includes 2 cases closed unsuccessfully.

Social workers also assessed whether youth are reintegrated to school, work, and community. Youths were assessed to be engaged in school if they have resumed attending school physically, and engaged in work if they were currently working at least part-time or engaged in internships or vocational training. Preliminary data indicated that 40% of youth overall were engaged in either school or work, with the percentage increasing to 83% for youths at Stage 4 of intervention (see Table 2). Our measure of community engagement is limited to youth's attendance of programs and activities conducted within our organization-overall, 24% of youth (50% at Stage 4 of intervention) have participated in interest-based activities (e.g., baking), vocational programs (e.g., career at the zoo exposure program), or animal-assisted activities.

## 7. Discussion

This model and its social-health approach to intervention has been found to be useful in targeting the hidden youth population in the Singaporean context.

### 7.1. Program strengths

#### 7.1.1. Multidisciplinary approach

The multidisciplinary approach adopted by HYIP blends the expertise of youth social work outreach and psychological medicine. By addressing hidden youth behavior as a multi-faceted phenomenon, the program provides youths and their families with integrated biopsychosocial services involving a multidisciplinary team, reducing the potential for dropout and overlap between multiple services.

However, adopting a multidisciplinary approach is not without its challenges. While involving different professionals provides a more holistic intervention, ensuring that services are coordinated is challenging for the social worker who usually serves as the overall case manager. Families are often confused about the role of each professional in the intervention plan. Convening case conferences with all professionals involved in the intervention *before* the family is engaged helps to delineate each party's role and align intervention goals. Regular communication between professionals also ensures that intervention goals are aligned, and that each party involved reinforces strategies and skills covered during therapy sessions. The social worker also functions as an intermediary, representing the voice of the family to communicate with other professionals when needed.

#### 7.1.2. Flexibility within the program structure

As highlighted by Adam's experience, intervention does not occur in a linear manner- youths may regress when setbacks or challenges arise. Unlike other programs, HYIP is not bound by fixed timelines or structure, allowing for back-and-forth movements between stages. Instead, HYIP professionals have the liberty to decide the mode of delivery, and when to cover specific treatment components. Another strength of the program is that it allows for a lengthy process of engagement and intermittent drop-out and re-entry as required. Such flexibility allows youths to progress at a comfortable pace. The integrative and long-term nature of the program also allows for thorough, evolving formulations of each case, as well as highly individualized intervention plans. Professionals are empowered to be as creative as possible with their outreach and youth engagement as they do not need to work within a fixed structure.

However, the flexible intervention approach coupled with the inherent difficulties engaging reclusive youth poses challenges for HYIP professionals. HYIP professionals faced difficulties managing their anxiety over the lack of progress in attaining intervention goals and felt frustrated when hidden youth regressed to an earlier stage of intervention after making initial progress. Frequent case supervision and joint case conferences to discuss challenges and brainstorm intervention approaches were identified as helpful strategies that enabled HYIP professionals to better manage their emotions about their cases and thus provide more effective intervention.

### 7.2. Limitations and future directions

There are also certain limitations of this program. Most notably, the development of HYIP was based on intervention models from Japan, Korea, and Hong Kong, as research on hidden youth is in its infancy in Singapore. The lack of evidence on local prevalence, trends, and characteristics of Singaporean hidden youth meant that the program could not account for these factors in its design, although it is known that certain factors unique to Singapore may affect hidden youth pathology and thus intervention. These include cultural factors, variation in parenting in a multi-racial society, and unique national military service conscription for males at age 18, at which time youths are required to leave home for enlistment.

Given the complexity and chronicity of the referred hidden youth, much of the success of engagement reported is dependent on a high level of clinical resource vested, professional commitment, responsive and contextualized clinical creativity, peer supervision, and tenacity of the outreach professionals. While the program's high resource investment helps to mitigate staff burnout and attrition risk, future collation of therapy evidence, streamlining clinical guidelines, and learning from identified successful approaches to engagement will help drive a value-based outcome.

There are some methodological constraints to this program. Currently, the success and good practices of HYIP are measured qualitatively through individual case studies reports. Moving forward, the program will build a system of standardized outcome measurement while recognizing the significant operational constraint in gathering data from reclusive hidden youth. Adopting a quantitative structured evaluation framework to determine the efficacy of this program would also help to mitigate against the small sample size and long duration of data collection.

Moving forward, the authors aim to address some of these limitations by working in collaboration with international and local practitioners to further develop good practices and adapt more established intervention frameworks. Specifically, we aim to incorporate additional intervention approaches, understand predictors for good response to intervention, and develop an evaluative framework to determine the effectiveness of HYIP. Taking reference from previous intervention studies with this population (24, 28), the following tools may be useful for evaluative purposes, and can be administered at intake and at regular 6-month intervals: For workers, the Clinical Global Impression Scale, and for caregivers: Revised Child Anxiety and Depression Scale (51). As no caregiver-report measures of hidden youth behavior are available in English, we aim to develop a tool that can be used in Singapore, based on the current Hidden Youth Questionnaire (52). As most youths were not able to complete any questionnaires during Stage 1, we aim to administer the following self-report questionnaires during Stage 2 instead, once some level of youth engagement has been achieved: the Beck Youth Inventory (45) and the Hikikomori Questionnaire 25 (44). Evaluation measures can be administered every 6 months, and at the point of case closure.

In addition, as there is a need for concerted effort to increase the evidence base of characteristics and trends of hidden youth in Singapore, we aim to work with relevant agencies to conduct a cross-sectional study of hidden youth to generate preliminary findings. To equip professionals working with youth with the knowledge to identify and refer cases to HYIP, we intend to organize a local conference, conduct training, and facilitate an interagency community of practice to foster collaboration and share best practices. We also hope to raise public awareness about hidden youth by encouraging youth who have successfully completed HYIP to share their stories on platforms such as social media or local news outlets. Our aim is to reduce stigma and empower hidden youth to come forward to seek support from HYIP.

## 8. Conclusion

This paper presents a social-health, multi-disciplinary framework to working with hidden youths in Singapore. Thus far, there is evidence through case studies of its efficacy, although more needs to be done to understand this unique population and implement evidence-based interventions.

## Data availability statement

The data presented in this article are not readily available as the dataset contains client data which is restricted due to ethical and privacy concerns. Further inquiries can be directed to the corresponding author, SK.

## Ethics statement

This studies involving human participants were reviewed and approved by Fei Yue Community Services Research Advisory Committee. Written informed consent to participate in the program and its evaluation was provided by the participants' legal guardian/next of kin.

## Author contributions

BY contributed to the conceptualization of the paper, the writing of the case study, reviewed the case study with Adam (not his real name), and Adam's caregiver and obtained informed consent. DL analyzed the service data, compiled findings from the reflective practice circles, and drafted the figures. SK, DL, and BY wrote the first draft of the manuscript. JW conceptualized the Hidden Youth Intervention Program (HYIP) with BY and SK and revised the manuscript. All authors contributed to manuscript revision, read, and approved the submitted version.

## Footnotes

^1^Programa Individualizado Psicoterapéutico para la Adicción a las Tecnologías de la información y la comunicación.
